# Changes in Primary School Children's Behaviour, Knowledge, Attitudes, and Environments Related to Nutrition and Physical Activity

**DOI:** 10.1155/2013/752081

**Published:** 2013-03-19

**Authors:** Anthea Margaret Magarey, Tahna Lee Pettman, Annabelle Wilson, Nadia Mastersson

**Affiliations:** ^1^Nutrition and Dietetics, Flinders University of South Australia, GPO Box 2100, Adelaide, SA 5001, Australia; ^2^Jack Brockhoff Child Health and Wellbeing Program, McCaughey VicHealth Centre for Community Wellbeing, Melbourne School of Population Health, University of Melbourne, 207 Bouverie Street, Carlton, VIC 3053, Australia; ^3^Discipline of Public Health, Flinders University of South Australia, GPO Box 2100, Adelaide, SA 5001, Australia; ^4^Health Promotion Branch, Department of Health, SA Health, P.O. Box 287 Rundle Mall, Adelaide, SA 5000, Australia

## Abstract

Rigorous evaluation of large-scale community-based obesity interventions can provide important guidance to policy and decision makers. The *eat well be active* (*ewba*) Community Programs, a five-year multilevel, multistrategy community-based obesity intervention targeting children in a range of settings, was delivered in two communities. A comprehensive mixed-methods evaluation using a quasiexperimental design with nonmatched comparison communities was undertaken. This paper describes the changes in primary school children's attitudes, behaviours, knowledge, and environments associated with healthy eating and physical activity, based on data from six questionnaires completed pre- and postintervention by students, parents, and school representatives. As self-reported by students in years from five to seven there were few significant improvements over time in healthy eating and physical activity behaviours, attitudes, knowledge, and perceived environments, and there were few changes in the home environment (parent report). Overall there were considerably more improvements in intervention compared with comparison schools affecting all environmental areas, namely, policy, physical, financial, and sociocultural, in addition to improvements in teacher skill and knowledge. These improvements in children's learning environments are important and likely to be sustainable as they reflect a change of school culture. More sensitive evaluation tools may detect behaviour changes.

## 1. Introduction

Evidence from evaluations of large-scale community-based interventions is building and suggests that modest improvements in child weight status can be achieved by multistrategy investments [1, 2]. Theoretical models guiding such interventions suggest that targeting behaviours, attitudes, knowledge, and skills at multiple levels, for example, individuals, teachers/leaders, and environment levels such as neighbourhood and school, environment types such as policy and culture, is likely to be most successful at slowing unhealthy weight gain [3–5]. Community-based, capacity-building approaches aim to promote sustainable skill development, thus improving environments that promote health outcomes [6]. The interaction between individuals' behaviour and their broader environments that influence eating and activity is complex, but recent systematic reviews of promising interventions suggest that core components of community-based programs aimed that school-aged children should include elements such as changes to school curriculum, number of physical activity sessions, food supply, workforce capacity building, and parental support, to develop competence in creating environments changes that support dietary and physical changes [7–10]. 

Although evidence is emerging to suggest that settings-based interventions targeting social and environment determinants have the potential to moderately improve some behaviours such as fruit and vegetable intake [11, 12], the sustainability of outcomes resulting from short-term interventions is still unknown. More analyses are needed for the changes in places where children live and learn, in relation to how conducive they are to healthy eating and physical activity. Such environments include physical (e.g., open space and equipment for play), policy (e.g., policy to reduce availability of energy-dense nutrient-poor food), sociocultural (e.g., teachers role modeling healthy behaviours), and financial environments (e.g., incentives and fundraisers to support healthy choices).

Interventions longer than one year are more likely to become embedded into school and parent activities, curriculum, and food supply than shorter interventions [12] and therefore hold more promise in improving attitudes, knowledge, and behaviours that contribute to healthy weight in the long term. Further evidence is seldom generated from pragmatic evaluations of practice; thus it is difficult to know whether the implementation and outcomes achieved in research projects are likely to be applicable and transferable beyond those contexts. Evidence generated through rigorous evaluation of large-scale community-based participatory implementation is likely to be most useful for informing decision-making on healthy eating and physical activity strategies and guidelines.

The *eat well be active *(*ewba*)* Community Programs* was a five-year community-based partnership approach of promoting environments for healthy eating and physical activity in children's settings in two communities in South Australia. A multilevel, multistrategy intervention was delivered, with the ultimate aim of promoting healthy weight through improved healthy eating and physical activity behaviours of children 0–18 years of age, via sustainable changes to physical, social, and policy environments. The purpose of this paper is to describe the changes in secondary outcomes, including primary school children's attitudes, behaviours, knowledge, and environments associated with healthy eating and physical activity in the home and school. To do so this paper draws on data from one component of the *ewba *evaluation: questionnaires completed by primary school students, their parents, teachers, and school principals. 

## 2. Methods

Ethics approval for this study was obtained from the Flinders University Social and Behavioural Research Ethics Committee and the ethics committees of SA Health, the Department of Education and Children's Services, and the Aboriginal Health Council of South Australia. 

The *ewba* initiative was funded for five years (2005–9) by the Health Promotion Branch of SA Health (Government of South Australia). The aim of *ewba* was to promote healthy weight of children and young people (0–18 years) and their families by working in partnership with a variety of settings to address both individual behaviour and environmental barriers and thus improve healthy eating and physical activity behaviours. Primary health care principles formed the basis for program development which was informed by multiple health promotion theories including a social ecological model and a community development approach. A set of guiding principles was developed, and these informed planning, development, implementation, and evaluation [13]. 

### 2.1. Program Development and Implementation

The programs were implemented in two South Australian communities: a metropolitan suburb (Morphett Vale) of a capital city with a population of approximately 23,000 and a rural city (the Rural City of Murray Bridge) with a population of approximately 18,000. Selection of the communities was based on total population size, relatively high socio-economic disadvantage [14, 15], higher than state-average prevalence of overweight, high proportions of Aboriginal and Torres Strait Islander people relative to the state average, and existing infrastructure and networks relevant to supporting the proposed project. 

Action plans were developed for each community based on the outcomes of a comprehensive community consultation process, involving over 500 community and agency stakeholders, and informed by the best available evidence and emerging recommendations. The intervention was multistrategy comprising numerous actions under six strategy types: workforce development and peer education, policy, infrastructure improvements, programs and resources, promotion and local marketing, and community development. The intervention was enacted across a number of key settings and stakeholder organisations where children and young people spend their time or receive services, including child care, education, and youth and community settings. 

One way in which the program's commitment to community development and intersectoral action was reflected was the formation of local action groups consisting of local stakeholders for each of the main age groups. These action groups supported implementation via on-going consultation, providing valuable advice, local expertise, ideas, and energy to the program. Further details of the consultation, action plan development, and interventions are available elsewhere [13, 16].

### 2.2. Program Evaluation

A comprehensive mixed-methods evaluation framework was designed. The methods comprising the evaluation framework have been described elsewhere [16–20]. The quantitative evaluation was a traditional quasiexperimental design with nonmatched comparison communities which did not receive the intervention. Evaluation occurred pre- and postintervention by repeat cross-sectional anthropometric measures and surveys. Comparison communities were chosen to match intervention communities as closely as possible according to population size, level of socioeconomic disadvantage, and proportion of Aboriginal and Torres Strait Islander people. 

#### 2.2.1. Questionnaires

A suite of 12 program-specific questionnaires was developed based on expert opinion and tools used from similar projects in other geographical locations to reflect the healthy eating and activity attitudes, environments, behaviours, and knowledge that were targeted in the intervention. Twelve questionnaires were developed. Data from six of these questionnaires are reported in this paper: child nutrition, child physical activity, teacher, parent, school principal, and school canteen manager questionnaires (Tables 2–7). For four of the questionnaires (child nutrition, child physical activity, teacher, and parent) individual items that represented a specific domain were condensed into “scores,” consistent with a social ecological framework. Scores covered similar domains across all questionnaires, including behaviour, attitude, knowledge, environment, and skills (specific to the teacher questionnaire). A target score was created that enabled comparison of the data to the target, based on healthy eating and physical activity guidelines [21, 22]. Score development is described elsewhere for the nutrition [23], parent and teacher [16], and physical activity [16] questionnaires. Most scores in the child nutrition questionnaire were shown to be reliable, and all behaviour scores were valid at the group level [23]. Unpublished data from the physical activity questionnaire demonstrated reliability for six of twelve scores with intraclass correlation coefficients (ICC) more than or equal to 0.5. Test retest reliability of the 14 scores in the parent questionnaire and the 12 scores in the teacher questionnaire, determined in 60 parents and 28 teachers, respectively, indicated intraclass correlation coefficients ranging from 0.37 to 0.92 (parent) (*P* < 0.05) and from 0.42 to 0.86 (teacher) (*P* < 0.05). 

Items in the principal and canteen manager surveys were classified using the ANGELO tool for analysis of environments related to obesity, that is, physical, policy, sociocultural, and financial (Tables 6 and 7) [5]. No psychometric testing has been performed on these questionnaires. 

#### 2.2.2. Data Collection

Baseline data were collected in 2006 (preintervention) and followup data in 2009 (postintervention). All primary schools in intervention and comparison sites were invited to participate (2006: *n* = 44, 2009: *n* = 45). The process of data collection in schools, including recruitment and logistics, has been reported [19]. In summary, a data collection team attended each participating school to take anthropometric measures (height, weight, and waist circumference (WC)) and administer two separate questionnaires about nutrition and physical activity to those students with parental consent and child assent, in school years from five to seven. Body mass index (BMI) was calculated (weight/height2) and BMI and WC *z*-scores and weight status determined using LMS growth Excel add-in (http://www.healthforallchildren.co.uk/). Further details have been reported [16, 19]. Prior to assessment day, teacher and principal questionnaires were sent to schools and collected by the data collection team. Parents who had provided consent to be contacted through their child's consent form were posted a questionnaire with a reply paid envelope for return to the research team. 

#### 2.2.3. Statistical Analysis

For the parent, teacher, and child questionnaires the proportion (%) meeting the target for each score was determined at baseline and followup in each of intervention (INT) and comparison (COMP) communities. An *χ* ^2^ test with the Monte Carlo 2-sided significance for proportions was used to determine the significance of the difference from baseline to followup in each of INT and COMP. For the principal and canteen manager questionnaires, the percentage of respondents reporting healthy eating and physical activity strategies in 2006 and 2009 was reported, and comparison across time was made using the exact test for 2 × 2 tables. 

## 3. Results

### 3.1. Participation Rates

Of the 44 schools invited to participate in the evaluation at baseline 39 (88.6%) agreed. The corresponding figures at followup were 45, 35, and 77.8%. The response rates for students, parents, principals, and canteen managers are shown in Table 1. The response rate for students was about five percent higher at baseline and followup in COMP schools compared with INT schools (2006: 44.7 versus 39.2%; 2009: 42.4 versus 37.5%) but was the same (50.4%) for all other surveys combined (Principal, teacher, and parents) in the two sites.

### 3.2. Student Surveys

#### 3.2.1. Weight

Anthropometric outcomes have been reported [16]. In brief, between baseline and followup assessments three years later, overweight/obesity prevalence and mean BMI *z*-score did not change significantly in INT or COMP. Waist circumference *z*-score decreased significantly in INT (−0.17, *P* < 0.05) but not in COMP (−0.10, *P* = NS); however, there was no significant difference between these changes (group by time interaction effect *P* = NS). 

#### 3.2.2. Behaviour Changes

Table 2 shows the number and percent of students meeting the target for each of the healthy eating and physical activity behaviour scores at baseline and followup in INT and COMP sites. It is important to note that less than 50% of students met the target score for noncore food, noncore beverages, and water and fruit intake, and less than 15% met the vegetable target score. At baseline over half the respondents met the target scores for being active in and out of school, but less than a fifth met the screen time recommendations. Overall there were similar changes across time in INT and COMP with few of these significant. The increases in those meeting the noncore food score and healthy behaviour scores are positive outcomes, as is the nonsignificant three-percent increase in those meeting the vegetable target score in INT. Decreases are seen in those meeting the water and fruit scores and three of the four activity scores. 

#### 3.2.3. Attitudes, Knowledge, and Environments

Table 3 shows the number and percent of students meeting the target attitude scores for fruit intake, vegetable intake, and physical activity, the proportion correctly identifying the number of daily serves of fruit (1-2) and vegetables (3–5) required for good health and the proportion meeting the target environment scores for fruit and vegetables and physical activity. 

At baseline, attitudes to fruit and physical activity were reasonably high. The only significant change was a decrease in the percent meeting the physical activity attitude score in both INT and COMP. 

There were significant increases in the proportion correctly identifying the number of fruit serves required for good health and nonsignificant increases for knowledge of vegetable serves. With respect to fruit serves the majority of incorrect answers were for more than 1-2 serves. A high proportion of children reported a supportive fruit and vegetable environment at baseline in both INT and COMP, and there was little change at followup. About half the students reported each of the school and home environments to be supportive of physical activity at baseline compared with only a third reporting the local environment to be supportive. Generally at followup a smaller proportion of students were reporting each of these environments to be supportive, but only the decrease in supportive local environment in COMP was significant. The initial proportion in COMP was high, and the proportion at followup was similar to INT schools. 

### 3.3. Home Environment (Parent Survey)

Table 4 shows the number and percent of parents who met the target scores indicative of a home environment supportive of healthy eating, physical activity, and appropriate small screen time. Food availability was estimated from food purchasing frequency, and while most parents met the target score for fruit and vegetables, only a third met the target for noncore foods and less than 10% for sweetened beverages at baseline. Overall changes at followup were small. Attitudes to healthy eating were similar in INT and COMP at baseline with small nonsignificant increases at followup. The proportion of parents meeting the target score for rules about healthy eating was high at baseline in both communities (approximately 70%) with little change in either community over time. Parental knowledge of both fruit and vegetable serves increased significantly in INT, but only knowledge of fruit serves increased significantly in COMP.

Parental attitudes and rules relevant to physical activity were low at baseline with only a third meeting the target scores for each of these. A significant increase in the percent meeting the target for attitudes was observed in COMP only; all other changes were minor and nonsignificant. 

### 3.4. School Environment

The teacher responses provide an indication of the classroom environment, and the school principal and canteen manager responses describe the wider school environment to which children are exposed. Table 5 shows the number and percent of teachers meeting the target for each of the healthy eating and activity scores at baseline and followup and the change over time. The proportion of teachers meeting the target for child exposure to healthy eating and fruit and vegetables was very low at baseline in both INT and COMP and even lower at followup for healthy eating but significantly higher for fruit and vegetables in INT only. The increase at followup in the proportion of teachers meeting the target score for child exposure to physical activity approached significance in INT (*P* = 0.07), while there was a nonsignificant fall in COMP. Teacher healthy eating skills were low at baseline but increased significantly in INT while declining in COMP. In contrast the proportion of teachers meeting the physical activity skill target score was unchanged in INT but declined significantly in COMP. Overall at baseline teacher knowledge of recommendations was greater for fruit and vegetables than for activity and screen time. All changes in INT and half the changes were positive in COMP, but only the increase in those correctly identifying daily fruit serves in INT was significant. 

Table 6 shows information regarding the wider school environment with respect to healthy eating classified according to environment type, namely, policy, physical, financial, and sociocultural. These data were obtained from the questionnaires completed by school principals and canteen managers. Data are presented as number (percent) of schools responding to assist with comparison, but the small numbers mean a change in one school may equate to between five and eight percent.  Table 7 shows similar information with respect to physical activity, obtained from the questionnaire completed by school principals. 

Overall changes from baseline to followup in the healthy eating environment of INT schools were positive. In contrast there were fewer positive changes in COMP schools. Of note is the decline in the proportion of schools having a healthy eating policy and using strategies to communicate healthy eating to parents. With respect to physical activity differences between changes in INT and COMP over time were similar with more positive and stronger changes in INT than in COMP. 

## 4. Discussion

This evaluation of a five-year community-based participatory intervention has demonstrated limited impact upon proximal indicators of child behaviours, attitudes, and knowledge, but very promising improvements in children's learning environments (namely schools) as reported by educators, leaders, and other key staff. A suite of surveys assessed children's behaviours, attitudes, knowledge, and environments through self-report, parent report (home environment), and principal, teacher, and canteen staff report (school environment). Individual items were combined as scores to align with evidence-based public health recommendations.

Overall there were considerably more improvements in INT compared with COMP schools affecting all environmental areas of policy, physical, financial, and sociocultural with respect to healthy eating and physical activity. The importance of these changes is that they are likely to be sustainable as they reflect a change of philosophy, and thus these positive environmental elements will be present for subsequent children entering the school. Of note however is the considerable opportunity for further improvements; for example, less than half of the INT schools had a healthy eating policy or physical activity policy at followup.

While there were few significant improvements over time in healthy eating and physical activity behaviours, attitudes, knowledge, and perceived environments in INT communities, and changes were often similar to those in COMP communities, these data provide an important insight into the overall levels of these factors in these communities. Clearly there is a significant scope for improving child behaviours particularly in the key areas of noncore food intake with less than a third meeting the target score and vegetable intake with less than 15% meeting the target score. Similarly less than one fifth met the recommendations for screen time.

The lack of significant change in child behaviours is not entirely unexpected as the intervention was multistrategy and comprised numerous actions only some of which were programs that directly involved children. Further child behaviours, attitudes, and overall environments for both healthy eating and activity are determined by factors at school, home, and the wider community. Overall, our finding of limited change in child behaviours is consistent with recent narrative reviews and meta-analyses of school-based healthy eating and physical activity programs targeting obesity-related behaviours, in that efficient interventions do not only aim at environmental changes [24] but also engage individuals directly. This may include educational components such as classroom curriculum and even computer-tailored personalised education [25]. Further, authors of such interventions similar to *ewba *have recently suggested that future obesity prevention strategies should target not only individuals and environments but also the household environment and family practices [26].

While the intervention included many strategies targeting schools, the principal means of influencing parents and the home environment was via the environment, that is, communication from schools. This explanation is in part supported by the changes in teacher and parent scores for healthy eating. There were several significant improvements in child exposure at school and teacher skills, attitude, and knowledge but few changes in parent scores. While parent knowledge of fruit serves increased, those who gave incorrect answers were more likely to overestimate requirements highlighting the gap between knowledge and behaviour as less than 50% students met the recommended fruit intake score. Based upon the recently published evaluations of similar interventions, we acknowledge the importance of home environments as a contributor to change in child weight status, compared with the influence of changes at the individual (child) level and school environment [26]. The findings with respect to improvements in teacher skills/attitude and knowledge around healthy eating and fruit and vegetables in INT communities are encouraging and expected, given the strong professional development focus of the *ewba *intervention, through providing a range of nutrition-related training sessions and support for teaching staff covering topics such as the state's healthy eating guidelines for schools and integrating healthy eating across the curriculum. While skills/attitude to physical activity did not change, there was a significant decline in COMP communities suggesting the intervention enabled maintenance of the baseline level. Concerning however is the low level of knowledge with respect to activity and screen time guidelines in both INT and COMP. 

It is difficult to make valid comparisons with other studies as there are few interventions that have taken such a broad approach and even fewer that have reported such a comprehensive range of outcomes, and the tools used to evaluate these outcomes vary. The finding of improved teacher skills/attitude and knowledge around healthy eating and fruit and vegetables is consistent with a recent intervention involving 300 students from six schools in low-income areas of Los Angeles, USA, where an intervention focusing on teacher training led to a positive change in teacher influence on students regarding fruit and vegetable attitudes, without demonstrating changes in student fruit and vegetable consumption [27]. The two-year APPLE intervention for 5–12-year olds, for example, targeted child activity and nutrition via activities provided by project activity coordinators in schools [28]. There were a few improvements in dietary intake in intervention children relative to controls (carbonated drinks, fruit juice, and fruit), no change in television viewing and conflicting results for physical activity. The four-year Romp and Chomp program in preschoolers which targeted community capacity building and environmental change also reported improvements in consumption of packaged snacks, fruit juice, and fruit and vegetables at followup in the intervention children but no changes in mean minutes viewing TV/DVD or in number of visits to the playground/park [2]. These evaluations identify that behavioural changes at the population level are generally small.

There are several considerations when interpreting the outcomes reported here. First the baseline surveys were conducted 7–9 months, after implementation had begun in 2006; so it is possible these positive findings are an underestimate for INT schools given that policy changes were among the first strategies implemented in INT schools. Other recent large-scale interventions involving a focus on food policy have demonstrated improvements in student behaviours and food choices after a three-year intervention [29]; thus it is reasonable to suggest that there is potential for food choice behaviour improvement in the *ewba *communities in future, with sustained healthy eating policies. Second the improvements observed in COMP are likely to be due to the changing political context in South Australia, where statewide nutrition-promoting policies began to be rolled out across all public schools from 2008. Thus the intervention was occurring against a background of community change. 

Although the findings of limited changes in child behaviour, attitude, and knowledge overall and between INT and COMP are consistent with current evidence, our findings may be attributed in part to the low sensitivity of the measurement tools to detect small changes over time. The availability of tools that measure trends in populations or the effectiveness of interventions related to nutrition and healthy eating, and of those identified, most were not sufficiently valid and reliable [29]. Hence, measuring the change in interventions such as the *ewba *Community Programs is problematic. It is also possible that the lack of changes is attributable to, in part, the time taken required to bring about change in child healthy eating and activity behaviours and that more time (in excess of the three years of evaluation followup in this project) is required to bring about changes that can be observed and measured. This is possible; considering previous research has identified that obesity interventions targeting multiple settings (including community, school, and home, like *ewba*) may enhance the impact and sustainability of obesity prevention efforts [30]. 

The changes in policy, physical, sociocultural, and financial environments are encouraging, particularly given that recent research has continued to highlight the importance of changing food and physical activity environments to improve related behaviours [29, 31]. Although the sample sizes were small due to the number of sites involved (thus statistical significance was unable to be measured) a majority of schools involved in the ewba intervention are represented in the evaluation surveys. The responses support actual school participation in healthy eating and physical activity policy and environment changes.

The evaluation of this community-based participatory project has a number of strengths that lend support to the findings of improved healthy environments for children. Although the comparison was nonrandomised, the selected comparison community with matched demographic characteristics accounts for secular changes observed throughout the intervention. The repeat cross-sectional design helped maintaining a pragmatic evaluation design, by capturing individual and school observations in context, rather than tracking changes within individuals longitudinally which is more intrusive and intensive. The sample size and response rates across all surveys were very good considering the pragmatic nature of the evaluation. First, similar proportions of students, parents, school principals, teachers, and canteen managers in INT and COMP responded at followup and baseline, giving confidence that respondents were representative of the broader population, thus reducing potential selection bias. That can occur in attrition. Second, psychometric testing of some of the survey tools has been completed or is underway [16, 23], confirming the appropriateness of the tools in obtaining accurate, repeatable data upon which conclusions have been drawn. Third, the multilevel data collection complements and helps triangulating the positive findings reported here, as not only can positive changes be observed across different levels (individual and environmental), but it has also been observed that child anthropometric measures improved more in INT compared with COMP in this community-based intervention [16]. 

The modest findings across multiple levels are considered encouraging taking into consideration the sheer complexity of conducting such a broad evaluation, specifically the assessment, interpretation, and reporting of the multiple factors that contribute to and shape child behaviour, and the importance of relevant tools that are accurate and sensitive to detect change. These issues underscore and reinforce the multitude of factors that must be considered when evaluating community-based efforts to modify the determinants of childhood obesity, in a pragmatic way. 

## Figures and Tables

**Table 1:** Number of eligible participants from the consenting schools and the number and proportion of those completing a survey at baseline and followup.

	Baseline 2006, 39 schools			Followup 2009, 35 schools		
	Possible	Completed	Response	Possible	Completed	Response
Students	3642^#^	1732	47.6^Φ^	3087^#^	1272	41.9^Φ^
Parents	1519*	983	65.7	1108*	726	65.5
Principals	40	36	90.0	35	28	80.0
Teachers	667	286	42.9	457	216	47.3
Canteen managers	29^*π*^	26	89.7	24^*π*^	19	79.1

^#^Enrolments as reported by the school principal.

*Those returning an affirmative consent form.

^*π*^Not all schools had a canteen.

^Φ^Consent rate 2-3% higher than response rate.

**Table 2:** Number (%) of primary aged school students meeting target scores at baseline and followup for healthy eating and physical activity behaviours by intervention (INT) and comparison (COMP) communities and percent change over time.

	Target score	Baseline (2006)		Followup (2009)		Change %	
		INT	COMP	INT	COMP	INT	COMP
Healthy eating	*N*	872	858	633	641		

Noncore food	<1	226 (25.9)	172 (20.0)	206 (32.5)	165 (25.7)	6.6*	5.7*
Noncore beverages	<1.3	303 (34.7)	286 (33.3)	228 (36.3)	228 (35.6)	1.6	2.2
Water	4	346 (39.7)	398 (46.4)	232 (36.7)	262 (40.9)	−3.0	−5.5*
Fruit	>6	400 (45.9)	385 (44.9)	262 (41.4)	261 (40.7)	−4.5	−4.2
Vegetable	>8	106 (12.2)	118 (13.8)	96 (15.2)	94 (14.7)	3.0	0.9
Healthy behaviour	>18	720 (82.6)	723 (84.3)	555 (87.7)	572 (89.2)	5.1*	5.0*

Physical activity	*N*	873	860	632	640		

Active at school	>7	600 (68.7)	662 (77.0)	414 (65.5)	456 (71.3)	−3.2	−5.7*
Active outside school	>4	547 (62.7)	607 (70.6)	410 (64.9)	442 (69.1)	2.2	−1.5
Active travel	>3	314 (36.0)	456 (53.0)	206 (32.6)	318 (49.7)	−3.4	−3.3
Total screen time	<18	176 (20.2)	164 (19.1)	117 (18.5)	110 (17.2)	−1.6	−1.9

*Significant change (*P* < 0.05) from baseline to followup.

**Table 3:** Number (%) of primary aged school students meeting target scores for healthy eating and physical activity attitudes, knowledge, and supportive environment scores at baseline and followup by intervention (INT) and comparison (COMP) communities and percent change over time.

	Target score	Baseline (2006)		Followup (2009)		Change %	
		INT	COMP	INT	COMP	INT	COMP
Healthy eating	*N*	872	858	633	641		

Attitude							
Fruit	≥16	647 (74.2)	648 (75.5)	483 (76.3)	473 (73.8)	2.1	−1.7
Vegetable	≥16	421 (48.3)	434 (50.6)	319 (50.4)	340 (53.0)	2.1	2.5
Knowledge							
Fruit serves^#^	2	312 (35.8)	311 (36.2)	286 (45.2)	283 (44.2)	9.4*	8.1*
Vegetable serves^#^	3	485 (55.6)	493 (57.5)	405 (64.0)	402 (62.7)	8.4	5.3
Environment							
Fruit and vegetable	≥19	762 (87.4)	732 (85.3)	536 (84.7)	532 (83.0)	−2.7	−2.3

Physical activity	*N*	873	860	632	640		

Attitude							
Physical activity	≥23	538 (61.6)	538 (62.6)	363 (57.4)	378 (59.1)	−4.2*	−3.5*
Environment							
Supportive school	≥19	412 (47.2)	464 (54.0)	289 (45.6)	333 (51.9)	−1.6	−2.1
Supportive local	≥7	262 (30.0)	363 (42.2)	211 (33.4)	220 (34.4)	3.4	−7.8*
Supportive home	≥8	607 (59.5)	479 (55.7)	349 (55.2)	339 (53.0)	−4.2	−2.7

*Indicates significant change (*P* < 0.05) in INT or COMP from baseline to followup, χ^2^ test with the Monte Carlo 2-sided significance.

^#^Daily serves required for good health for child aged 9–11 years based on the Australian Guide to Healthy Eating (AGHE) [22]: fruit 1-2, vegetable 3–5.

**Table 4:** Number (%) of parents meeting the target scores for healthy eating and physical activity at baseline and followup by intervention (INT) and comparison (COMP) communities and percent change over time.

	Target score	Baseline (2006)		Followup (2009)		Change	
		INT	COMP	INT	COMP	INT	COMP
*N* ^#^		499–517	434–457	326–346	360–374		
Healthy eating							
Food availability							
Sweet beverages	≥12	46 (9.2)	39 (9.0)	53 (16.3)	35 (9.7)	7.1	0.7
Noncore foods	≥21	181 (35.2)	124 (28.1)	127 (36.7)	93 (25.3)	1.5	−2.8
Fruit and vegetables	≥12	473 (91.3)	396 (87.8)	311 (89.9)	339 (90.6)	−1.4	2.8
Attitudes	≥24	187 (36.7)	177 (39.3)	140 (40.8)	152 (41.4)	4.1	1.5
Rules	≥40	354 (70.4)	301 (67.6)	241 (71.5)	240 (66.1)	1.1	−1.5
Knowledge of healthy eating^∧^							
Fruit serves	1-2	311 (61.3)	268 (60.3)	227 (71.3)	248 (67.5)	10.0*	7.2*
Vegetable serves	3–5	326 (64.2)	295 (66.2)	248 (73.9)	248 (67.5)	9.7*	1.3
Activity							
Attitude	≥28	134 (26.1)	155 (34.4)	92 (27.1)	168 (45.3)	1.0	10.9*
Rules	≥12	183 (35.4)	171 (37.4)	119 (34.8)	135 (36.4)	−0.6	−1.0

^#^Number of responses varies between items.

*Significant difference between baseline and followup within condition.

^∧^Proportion identifying correct answer as per the Australian Guide to Healthy Eating guidelines serves per day [22].

**Table 5:** Number (%) of teachers meeting the target scores for healthy eating and physical activity at baseline and followup by intervention (INT) and comparison (COMP) communities and percent change over time.

	Target score	Baseline (2006)		Followup (2009)		% Change	
		INT	COMP	INT	COMP	INT	COMP
N^∧^		129–137	124–129	98–106	84–98		
Child exposure							
Healthy eating	≥15	20 (14.9)	11 (8.5)	10 (9.4)	3 (3.4)	−5.5	−5.1
Fruit and vegetables	≥16	15 (7.6)	15 (12.1)	18 (17.0)	11 (11.6)	9.4**	−0.5
Physical activity	≥30	34 (25.6)	39 (30.7)	39 (37.1)	23 (24.7)	11.6	−6.0
Water	Allow in class	111 (81.1)	110 (85.1)	97 (91.2)	85 (86.9)	10.1**	1.8
Teacher skills/attitude							
Healthy eating	≥7, ≥16^#^	8 (6.2)	15 (11.7)	19 (19.4)	3 (3.4)	13.2**	−8.3
Fruit and vegetables	≥24	44 (32.1)	51 (39.5)	45 (42.5)	37 (41.1)	10.4	1.6
Physical activity	≥27, ≥36^#^	32 (24.1)	37 (29.1)	20 (20.0)	6 (7.1)	−4.1	−22.0**
Teacher knowledge*							
Fruit serves	1-2	81 (55.5)	78 (56.1)	83 (73.5)	67 (67.0)	18.0**	10.9
Vegetables serves	3–5	115 (78.8)	105 (75.5)	96 (85.0)	75 (75.0)	6.2	−0.5
Physical activity mins	≥60	72 (49.3)	57 (41.0)	64 (56.6)	51 (51.0)	7.3	10.0
Screen time mins	<120	38 (26.2)	45 (32.4)	31 (27.7)	28 (28.0)	1.5	−4.4

^∧^Number of responses varies between items.

*Proportion identifying correct answer. Fruit and vegetables: Australian Guide to Healthy Eating guidelines serves per day (Smith et al. [22]); Physical Activity and Screen time recommendations minutes per day [21].

**Significant difference between baseline and followup within condition.

^#^Higher target refers to 2009 which included four years for professional development.

**Table 6:** Number (%) of primary schools reporting healthy eating strategies in 2006 and 2009, by intervention or comparison, and percent change over time based on responses from the principal questionnaire except where indicated otherwise.

Environment	Baseline (2006)		Followup (2009)		Change (%)	
	INT	COMP	INT	COMP	INT	COMP
*N*	22	14	15	13		
Policy						
Have a healthy eating policy (n, (%))	6 (27)	4 (29)	6 (43)	1 (8)	+16*	−21
Policy items that were met “completely” (for all centres who had a healthy eating policy)	61.5%	53.7%	84.1%	48.1%	+23	−6
Physical						
Canteens sold selected healthy food products “every day they are open”^†^	7 (43)	4 (42)	4 (38)	4 (42)	−5	−1
Canteens “NEVER” sold selected unhealthy food products^†^	9 (56)	4 (35)	7 (66)	6 (68)	+10	+33*
Canteens introduced new healthier products^†^	8 (53)	5 (47)	3 (33)	1 (15)	−20*	−32
Two biggest food sellers were healthy choices, classified by Right Bite colour spectrum (green category foods)^†^	3 (19)	5 (46)	4 (40)	5 (53)	+21*	+7
Have set fruit/vegetable “break” during class time	11 (68)	8 (79)	9 (93)	8 (92)	+25*	+13*
Financial						
Never use any unhealthy foods^#^ for fundraising	9 (39)	10 (69)	9 (61)	9 (69)	+22*	0
Use fruit and vegetables for fundraising	9 (42)	3 (23)	6 (42)	3 (23)	0	0
Sociocultural						
Reported no food rewards allowed	7 (32)	5 (36)	12 (80)	7 (50)	+48*	+14*
Strategies used more than once/term to communicate healthy eating to parents	4 (18)	5 (38)	7 (47)	2 (15)	+28*	−23

^†^Canteenmanager questionnaire.

^#^Biscuits/cakes/lamingtons; confectionary/chocolates/lollies.

*Significant difference between baseline and followup within condition.

**Table 7:** Percent of primary schools reporting physical activity strategies in 2006 and 2009, by intervention or comparison, and change.

Environment	Baseline (2006)		Followup (2009)		Change (%)	
	INT	COMP	INT	COMP	INT	COMP
*N*	22	14	12	13		
Policy						
Have a physical activity policy (*n*, (%))	6 (27)	2 (14)	5 (42)	2 (15)	+14*	+1
Policy items that were met “completely” (for all schools who had an active play/physical activity policy)	44%	53%	35%	18%	−9	−35
Physical reported by principals						
Provide noncompetitive PA options	14 (64)	9 (62)	10 (87)	13 (100)	+23*	+38*
Provide organised PA more than once/week at set times before school; during school; after school; break times	6 (28)	3 (21)	4 (35)	3 (22)	+7	+1
Sociocultural						
Approaches used to promote PA (selected from a list of 8 possible approaches)	10 (44)	7 (52)	6 (52)	7 (54)	+8	+2
Strategies used more than once/term to communicate physical activity to parents	3 (14)	6 (46)	4 (33)	5 (38)	+20*	−8

*Significant difference between baseline and followup within condition.
